# Supplementary material for: The construction process for pre-stressed ultra high performance concrete communication tower
Source: PLoS One. 2020 Nov 4;15(11):e0238654. doi: 10.1371/journal.pone.0238654 (PMC7641448; doi:10.1371/journal.pone.0238654)
Supplement: S1 File — (PDF) [file pone.0238654.s001.pdf]

Additional Calculation and information

For Review - Not for Publish

1. Section Properties

TheUHPC tower segment is a pretension precast tapered pipe made from steel fiber reinforced ultra-high performance concrete (UHPC), thus **transformed** sectional properties will be used in the following calculation.

1.1 Material Properties

The following present the material properties of UHPC both at transfer and at service stages.

	**At transfer (t =2 days)**	**At service (t = infinity)**
Characteristic Cube Compressive Strength, f_{Ucu} (MPa)	85	165
Characteristic Cyl. Compressive Strength, f_{Ucu} (MPa)	80	160
Characteristic Compressive Strength, f_{Uck} (MPa)	70	150
Density of Concrete (kg/m^3)	2450	2450
Characteristic Elastic Tensile Strength, $f_{U,tek}$ (MPa)	4<br>0 (at joint)	7.0<br>0 (at joint)
Characteristic Ultimate Tensile Strength, $f_{U,tuk}$ (MPa)	4<br>0 (at joint)	7.0<br>0 (at joint)
Modulus of Elasticity, E_U (GPa)	40	50
Poisson's Ratio	0.2	0.2

The following present the material properties of prestressed strand used.

Type	**S15**
Diameter (mm)	15.2
Density of Steel (kg/m^3)	7840
Type	Low Relaxation
Nominal Section (mm^2)	140
Nominal Weight (kg/m)	1.10
Specified Breaking Load (kN)	260
Specified Load at 1% Elongation (kN)	235
Modulus of Elasticity, E_p (GPa)	195
n_p	4.88 (at transfer)<br>3.90 (at service)

The following present the material properties of steel reinforcement used.

	**Steel Reinforcement Grade460 (BS4449)**					
**Type**	**T10**	**T12**	**T16**	**T20**	**T25**	**T32**
Dia. (mm)	10	12	16	20	25	32
Density of Steel (kg/m^3)	7840	7840	7840	7840	7840	7840
A_s (mm^2)	79	113	201	314	491	804
E_s (GPa)	200	200	200	200	200	200
σ_{sy} (MPa)	460	460	460	460	460	460
ε_{sy}	0.002	0.002	0.002	0.002	0.002	0.002
F_{sy} (kN)	36	52	92	115	226	370

1.2 Transformed Section Properties of Girder

The section modulus ratios for strands are taken as $n_p = E_p / E_c$. Thus at transfer and service the n values are:

- $n_{transfer}$ = 195/40 = **4.88**$n_{service}$ = 195/50 = **3.9**

At transfer and Service

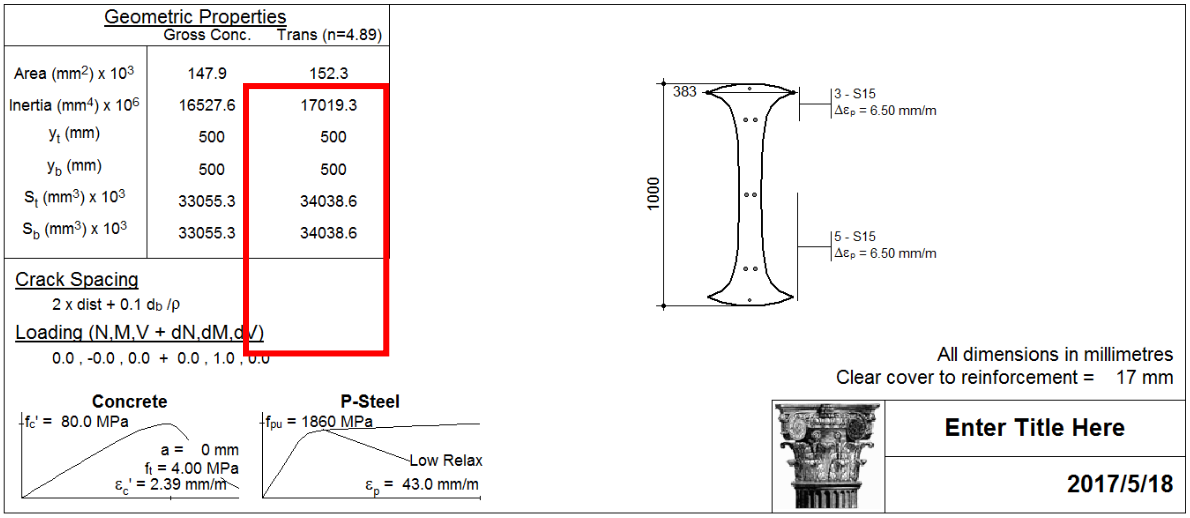

At Transfer

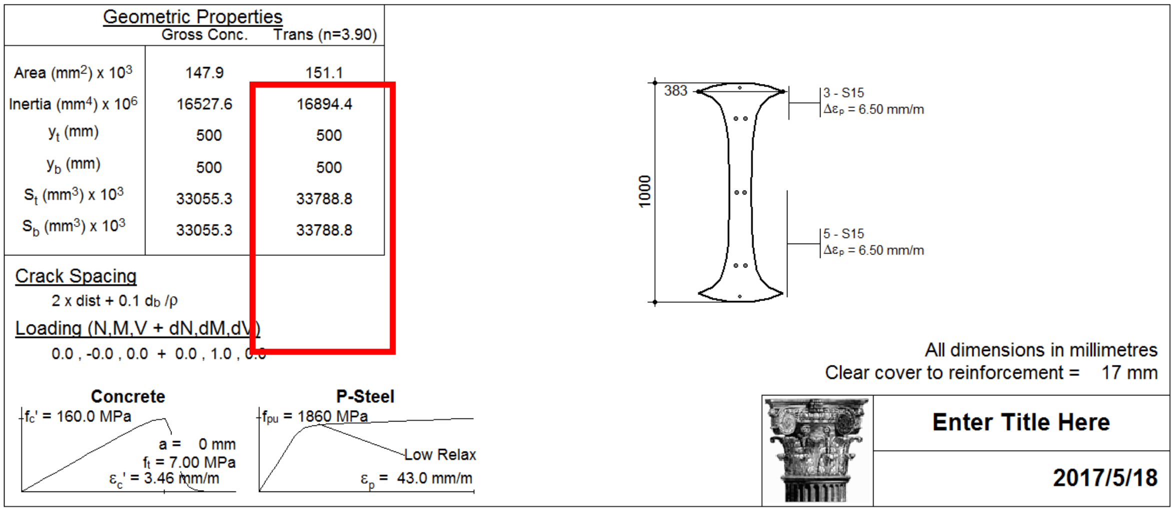

At Service

	At transfer (t = 2 days)	At service (t = infinity)
n	4.33	3.9
A (mm^2)	152,300	151,100
I_{xx} (x 10^9 mm^4)	17.02	16.89
y_t (mm)	500	500
y_b(mm)	500	500
Z_t (x 10^6 mm^3)	34.04	33.79
Z_b (x 10^6 mm^3)	34.04	33.79

2. Serviceability Calculation

2.1 At Transfer

Assume all the PT strands prestress to 75% of its breaking load (i.e. 260 kN) and the strands immediately have 5% of losses during transfer. Besides, the long term losses is taken as another 10% where the pipe has undergone all time-effect losses, such as creep and shrinkage and relaxation of the tendons.

Therefore the jacking force for each strands is P_j = 260 kN x 0.75 = 195 kN

The initial prestressed force P_i = 0.95 P_j = 0.95 x 195 = 185.25 kN (use at transfer, t =2 days)

The effective prestressed force P_e = 0.90 x P_i = 0.9 x 185.25 = 167 kN (use at service, t = infinity)

The concrete stress limits used are:

At transfer:

- 0.6 f_{Uck} = 0.6 x 70 = 42 MPa (for compression)
- f_{td} = $f_{U,etk}$ / γ_c = 4.0 / 1.0 = 4 MPa (for tension) at monolithic section

The extreme fiber stresses during transfer at the support regions(ends of beam) are:

$$\sigma_{top,transfer} = -\frac{\sum P_i}{A} + \frac{\sum P_i \ e_p}{Z_t} = -\frac{185.25 \times (8) \times 10^3}{152300} + \frac{0}{34.04 \times 10^6} \quad \text{(OK)}$$
$$= -9.731 + 0 = -9.731 MPa \ < \ -42 MPa$$

The resultant stress at the top flange is -9.73 MPain compression, which is less than the stress limit of the concrete in compression at transfer.

$$\sigma_{bot,transfer} = -\frac{\sum P_i}{A} - \frac{\sum P_i \ e_p}{Z_b} = -\frac{185.25 \times (8) \times 10^3}{152300} - \frac{0}{34.04 \times 10^6} \quad \text{(OK)}$$
$$= -9.731 + 0 = -9.73 MPa \ < \ 0.6 f_{Uck} = 42 Pa$$

The resultant stress at the bottom flange is -9.73 MPa in compression, which is a lot less than the stress limit of concrete in compression. In another word, UHPC prestressed pipe has plenty of reserve compression capacity and crushing of the concrete can never occur.

Therefore, the beam will not crack or crush at any section during transfer of the prestressing.

2.2 At Service

Assume all the PT strands prestress to 75% of its breaking load (i.e. 260 kN) and the strands immediately have 5% of losses during transfer. Besides, the long-term losses is taken as another 10% where the pipe has undergone all time-effect losses, such as creep and shrinkage.

Therefore the jacking force for each strands is P_j = 260 kN x 0.75 = 195 kN

The initial prestressed force $P_i = 0.95\ P_j = 0.95 \times 195 = 185.25$ kN (use at transfer, t = 28 days)

The effective prestressed force $P_e = 0.90 \times P_i = 0.90 \times 185.25 = 167$ kN (use at service, t = infinity)

The concrete stress limits used are:

<u>At service:</u>

- $0.6\ f_{Uck} = 0.6 \times 150 = 90$ MPa (for compression)
- $f_{td} = f_{U,etk} / \gamma_c = 7.0 / 1.0 = 7$ MPa (for tension) at monolithic section

The extreme fiber stresses during transfer at the support regions (ends of beam) are:

$$\sigma_{top,service} = -\frac{\sum P_e}{A} + \frac{\sum P_e\ e_p}{Z_t} = -\frac{167 \times (8) \times 10^3}{151100} + \frac{0}{33.79 \times 10^6} \qquad \text{(OK)}$$
$$= -8.84 + 0 = -8.84 MPa\ <\ -42 MPa$$

The resultant stress at the top flange is -9.73 MPa in compression, which is less than the stress limit of the concrete in compression at transfer.

$$\sigma_{bot,service} = -\frac{\sum P_e}{A} - \frac{\sum P_e\ e_p}{Z_b} = -\frac{167 \times (8) \times 10^3}{151100} + \frac{0}{33.79 \times 10^6} \qquad \text{(OK)}$$
$$= -8.84 + 0 = -8.84 MPa\ <\ -42 MPa$$

The resultant stress at the bottom flange is -8.84 MPa in compression, which is a lot less than the stress limit of concrete in compression. In another word, UHPC prestressed pipe has plenty of reserve compression capacity and crushing of the concrete can never occur.

Therefore, the pipe will not crack or crush.

To find the cracking moment of the pipe when the pipe is 1m diameter.

$$f_{Utek} = -8.84 + \frac{M_{cr}}{Z_b}$$

$$7 = -8.84 + \frac{M_{cr}}{33.79}$$

Therefore the cracking moment is 535 kNm.

3. Ultimate Strength Calculation

3.1 Design Moment Resistance (M_{Rd})

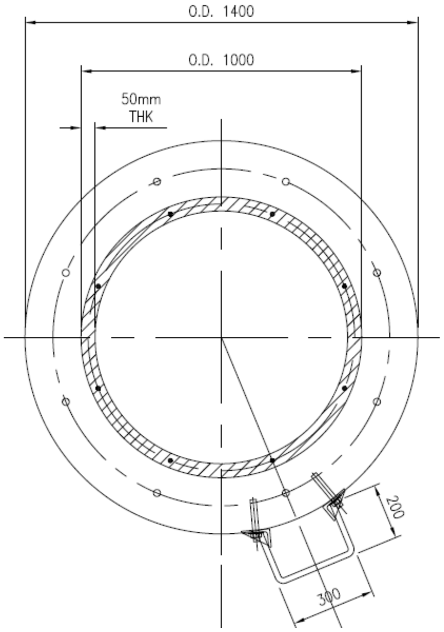

The design moment resistance can be easily calculated using the moment equilibrium method. First calculated the factored design tendon tension forces.

The material factor used for the strands is 1.15.

The material factor used for the UHPC is 1.3.

A rectangular stress block for the compression part is used assume in the top flange. The factored compressive stress can be taken as f_{Uck}/γ = 150/1.3 = 115 MPa. Then using force equilibrium

method. Sum of C must be equal to Sum of T. In this calculation the top tendon is ignored. The next step is to find X.

Response2000 gives a value of M_{Rd}= 838 kNm.

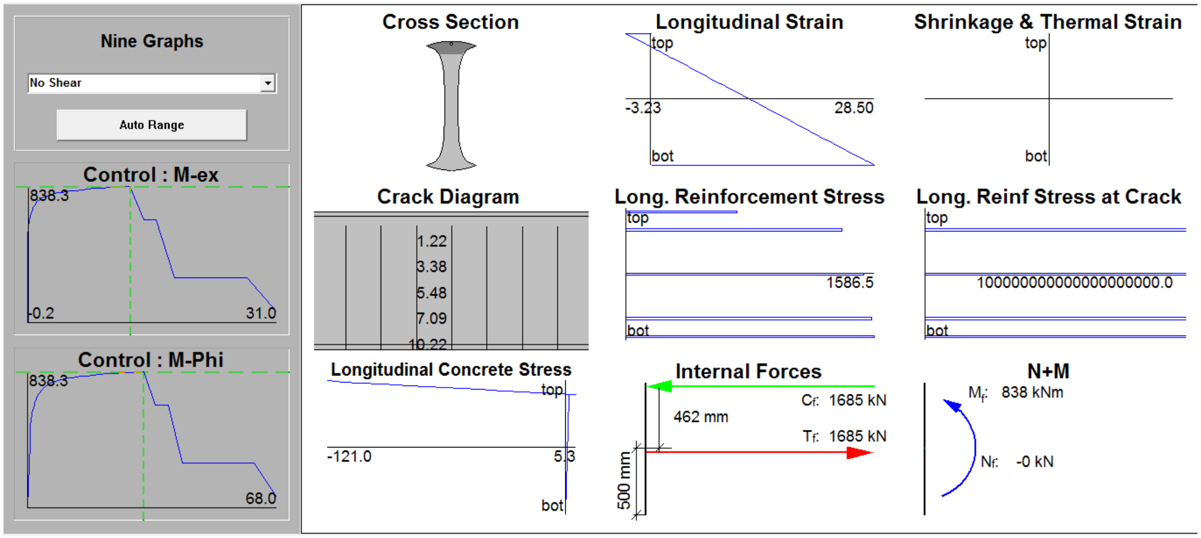

Design Drawings for Details

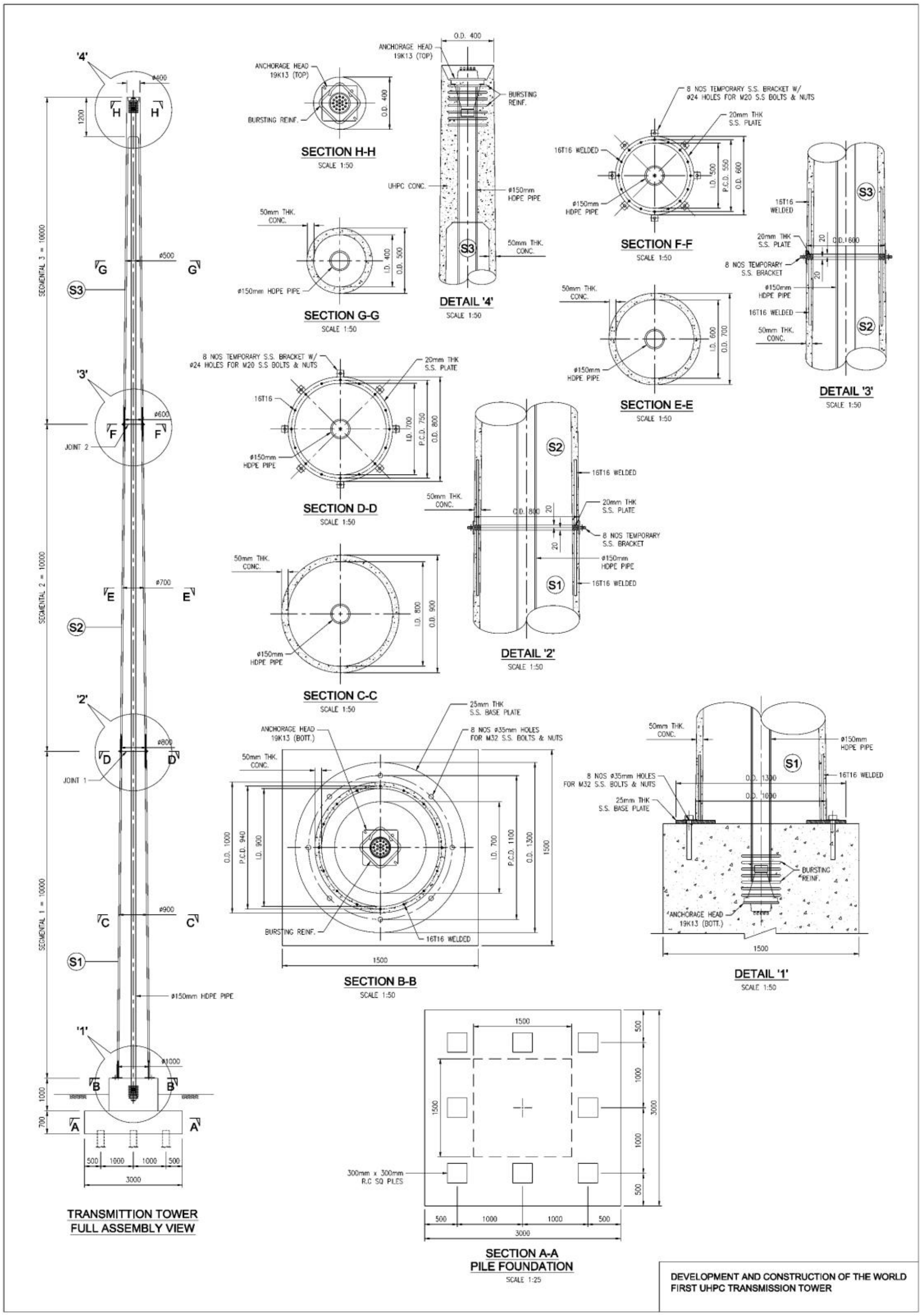

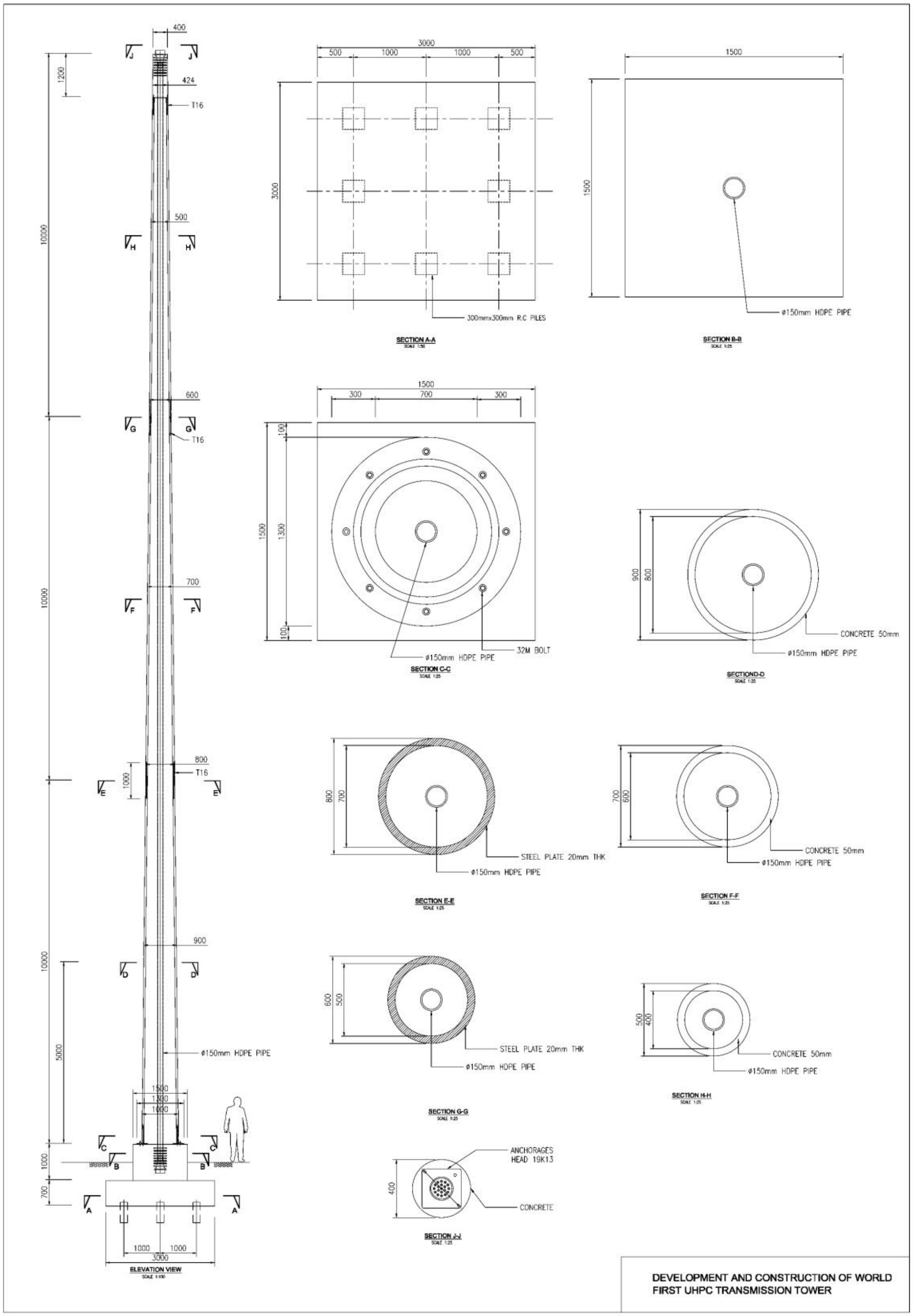
SECTION A-A
300mmx300mm R.C PILES
SECTION B-B
ø150mm HDPE PIPE
SECTION C-C
32M BOLT
SECTION D-D
CONCRETE 50mm
SECTION E-E
STEEL PLATE 20mm THK
SECTION F-F
SECTION G-G
SECTION H-H
SECTION J-J
ANCHORAGES HEAD 19K13
CONCRETE
ELEVATION VIEW
DEVELOPMENT AND CONSTRUCTION OF WORLD FIRST UHPC TRANSMISSION TOWER

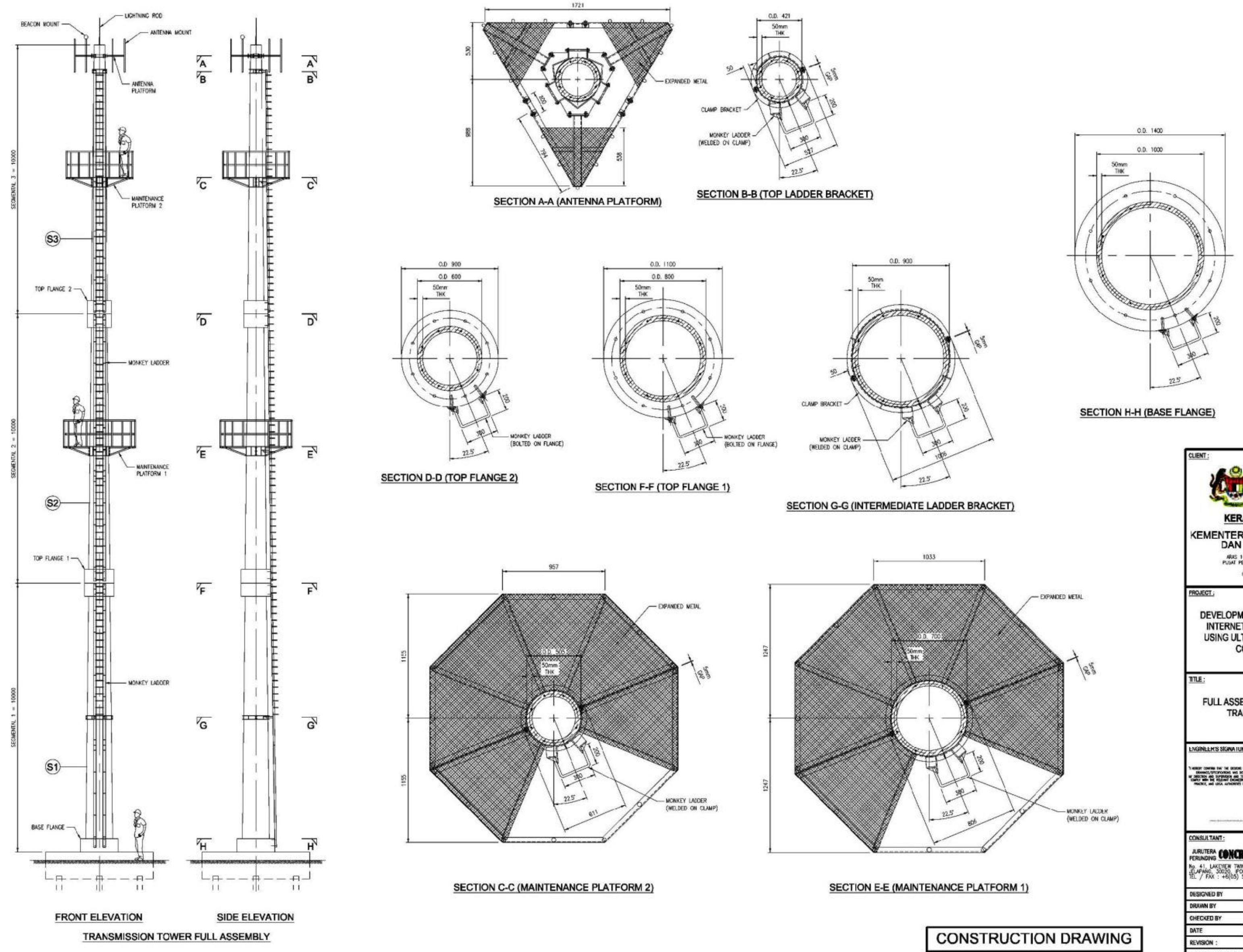

CONSTRUCTION DRAWING

CLIENT :

KERAJAAN MALAYSIA

KEMENTERIAN SAINS, TEKNOLOGI DAN INOVASI (MOSTI)

ARAS 1-7, BLOK C4 & C5, KOMPLEKS C, PUSAT PENTADBIRAN KERAJAAN PERSEKUTUAN, WILAYAH PERSEKUTUAN 62662 PUTRAJAYA, MALAYSIA

PROJECT :

DEVELOPMENT & CONSTRUCTION OF INTERNET TRANSMISSION TOWER USING ULTRA HIGH PERFORMANCE CONCRETE (UHPC)

TITLE :

FULL ASSEMBLY OF PRECAST UHPC TRANSMISSION TOWER

ENGINEER'S SIGNATURE :

CONSULTANT :

JURUTERA PERUNDING **CONCRETE TECHNOLOGY**

No. 41, LAKEVIEW TWIN VILLA, LINGKARAN MERU VALLEY 2A, JELAPANG, 30020, IPOH, PERAK, MALAYSIA.
TEL / FAX : +6(05) 5282813

DESIGNED BY	FAIZ	SCALE
DRAWN BY	DIANA	1 : 65
CHECKED BY	ADJ PROF IR DR VOO YEN LEI	
DATE	MARCH 2015	
REVISION :		

DRAWING NO.
MOSTI/JPCT/03/2015/TT/01

CONSTRUCTION DRAWING

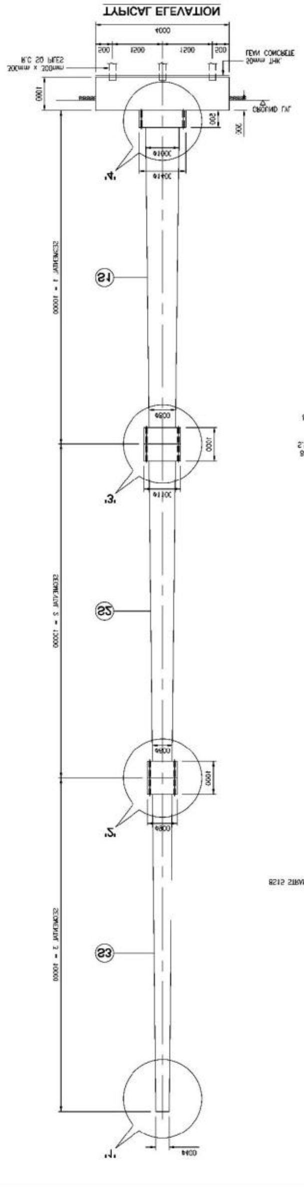

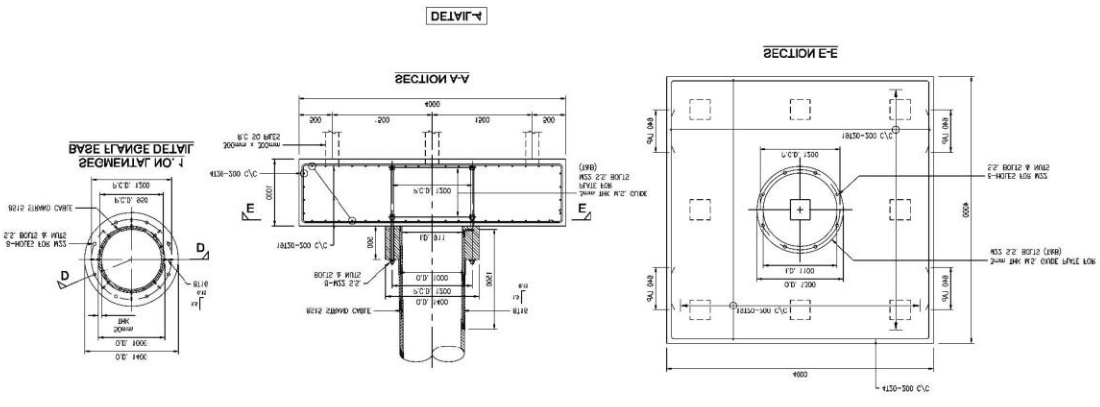

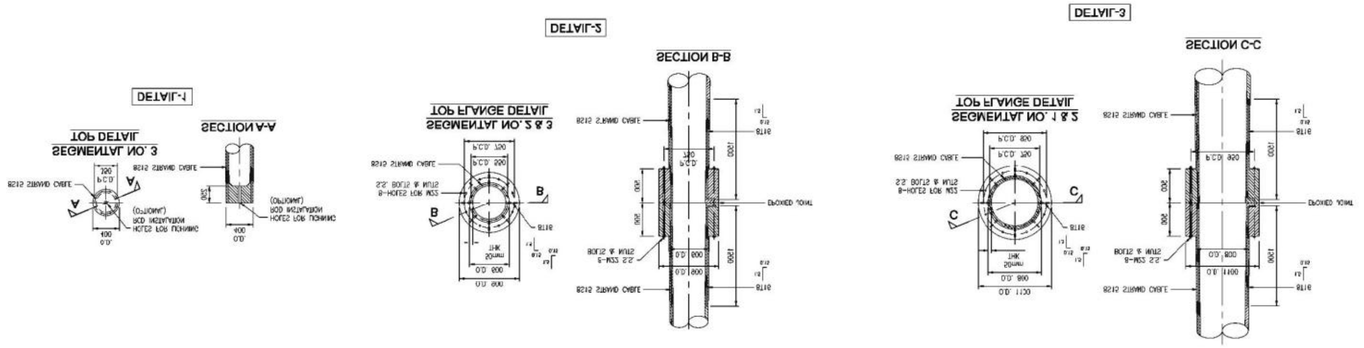

SPECIFICATION OF SPECIAL CONCRETE – ULTRA-HIGH PERFORMANCE CONCRETE (UHPC)

1. THE UHPC USED SHALL BE STEEL FIBER REINFORCED GRADE 120 CONCRETE. THE CONCRETE USED SHALL COMPOSE OF THE FOLLOWING RAW MATERIALS:
   a. ORDINARY PORTLAND CEMENT COMPLY WITH THE REQUIREMENT OF MS522-PART1.
   b. FINE SAND WITH MAXIMUM GRANULAR SIZE NOT LARGER THAN 1.5 mm AND COMPLY WITH THE REQUIREMENT OF ASTM C 33.
   c. DENSIFIED SILICA FUME (CEMENT REPLACEMENT SHALL NOT LESS THAN 10% AND SHALL NOT MORE THAN 25%) COMPLY WITH THE REQUIREMENT OF ASTM C 1240.
   d. STEEL MICRO-STEEL FIBER WITH TENSILE STRENGTH ABOVE 2200 MPa WHICH MANUFACTURED FROM HIGH CARBON STEEL WIRE COMPLY WITH THE REQUIREMENT OF DIN17223.
   e. POLYCARBOXYLATE BASED HIGH-RANGE WATER REDUCER WITH MIN 30% OF SOLID.
2. THE WATER-TO-BINDER RATIO OF THE CONCRETE MIX SHALL NOT MORE THAN 0.18.
3. THE AMOUNT OF STEEL FIBER INCLUDE IN THE CONCRETE MIX DESIGN SHALL NOT LESS THAN 1.5% BY VOLUME.
4. THE UHPC USED IN THE PRECAST ELEMENTS SHALL HAS 1 DAY AND 28 DAYS MEAN CUBE COMPRESSIVE STRENGTH OF 60 AND 120 MPa AND ABOVE, RESPECTIVELY, COMPLY TO REQUIREMENT OF BS. EN 12390-3 : 2009
5. THE UHPC USED IN THE PRECAST ELEMENTS SHALL HAS THE FOLLOWING 28 DAYS FLEXURAL TENSILE STRENGTH OF (COMPLY WITH BS EN 14651: 2005):
   a. MEAN VALUE OF MODULUS OF RUPTURE OF (f_{ct}) SHALL BE 30 MPa
   b. MEAN RESIDUAL FLEXURE STRENGTH AS DEFINED BY A MIDSPAN DISPLACEMENT OF 0.47mm (fR1) SHALL BE 30 MPa;
   c. MEAN RESIDUAL FLEXURE STRENGTH AS DEFINED BY A MIDSPAN DISPLACEMENT OF 2.17mm (fR3) SHALL BE 30 MPa;
6. THE UHPC USED IN THE PRECAST ELEMENTS SHALL BE WATER TIGHT. THEREFORE THE 28 DAYS MEAN WATER ABSORPTION SHALL BE LESS THAN 0.25% COMPLY TO BS 1881-PART 122, 1983.
7. THE UHPC USED IN THE PRECAST ELEMENTS SHALL HAVE SUPERIOR DURABILITY. THEREFORE THE RAPID CHLORIDE PERMEABILITY TEST (RCPT) VALUE SHALL NOT MORE THAN 300 COULOMBS COMPLY TO REQUIREMENT OF ASTM C1202-2005.

DRAWING NO.	MOSTI/JPCT/03/2015/TT/02	
REVISION :		
DATE	MARCH 2015	SCALE
CHECKED BY	ADJ PROF IR DR VOO YEN LEI	1 : 60
DRAWN BY	DIANA	
DESIGNED BY	FAZ	

CONSULTANT :
JURUTERA PERUNDING CONCRETE TECHNOLOGY
NO. 41, LAKEVIEW TWIN VILLA, LINGKARAN MERU VALLEY 3A, 30020 IPOH, PERAK, MALAYSIA.
TEL \ FAX : +6(05) 5282813

ENGINEER'S SIGNATURE :

TITLE :
TRANSMISSION TOWER
REINFORCEMENT DETAILS

PROJECT :
DEVELOPMENT & CONSTRUCTION OF INTERNET TRANSMISSION TOWER USING ULTRA HIGH PERFORMANCE CONCRETE (UHPC)

CLIENT :
KERAJAAN MALAYSIA
KEMENTERIAN SAINS, TEKNOLOGI DAN INOVASI (MOSTI)
ARAS 1-7, BLOK C4 & C5, KOMPLEKS C,
PUSAT PENTADBIRAN KERAJAAN PERSEKUTUAN,
WILAYAH PERSEKUTUAN
PUTRAJAYA, MALAYSIA

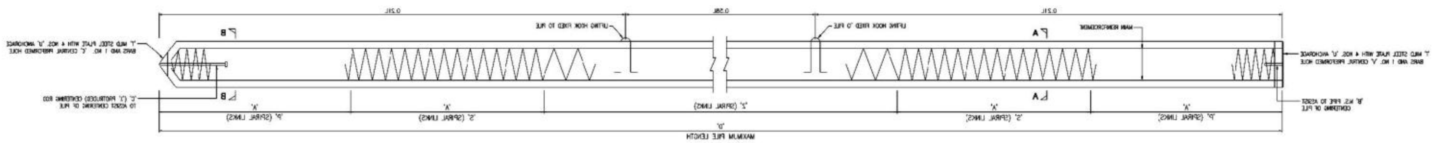

LONGITUDINAL SECTION OF TYPICAL INITIAL RC PILE

SCALE 1 : 30

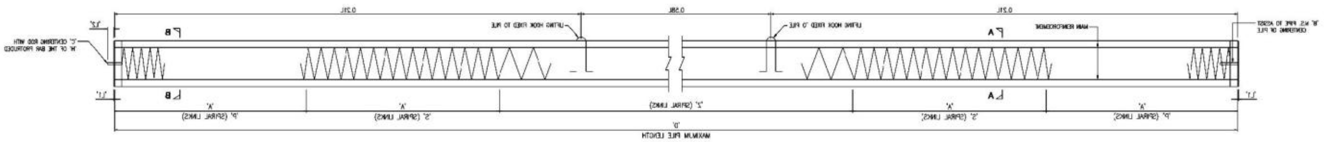

LONGITUDINAL SECTION OF TYPICAL EXTENSION RC PILE

SCALE 1 : 30

NOTES:-

1. PILE DESIGN ARE IN ACCORDANCE WITH BS 8110:1985 AND BS 8004:1986.
2. STEEL REINFORCEMENT SHALL CONFORM TO MS 146:1988.
3. CONCRETE MIX FOR PILE SHALL BE OF GRADE 45. THE MINIMUM CONCRETE STRENGTH AT 7 DAYS SHALL BE 32N/mm2 AND THE MINIMUM STRENGTH AT 28 DAYS SHALL BE 45N/mm2.
4. CONCRETE COVER TO THE LATERAL REINFORCEMENT SHALL BE 30mm.
5. THE MAXIMUM AGGREGATE SIZE SHALL BE 20mm AND SHALL CONFORM TO MS 29: 1971.
6. THE CEMENT USED SHALL BE ORDINARY PORTLAND CEMENT COMPLYING WITH MS 522: PART 2:1989.

PILE DIMENSIONS AND DETAILS

PILE NOMINAL SIZE (mm x mm)	MAIN REINFORCEMENT Nos. x Dia. (mm)	COVER (mm)	RECOMMENDED WORKING LOAD* (Tonnes)	A (mm)	B	C	D (mm)	E (mm)	F (mm)	G (mm)	H (mm)	J (mm)	K (mm)	W (mm X mm)	L_1 (mm)	L_2 (mm)	M	N	P	Q (mm)	R	S	T (mm)	U	V (mm)	Z (mm)
[illegible]	[illegible]	[illegible]	[illegible]	[illegible]	[illegible]	[illegible]	[illegible]	[illegible]	[illegible]	[illegible]	[illegible]	[illegible]	[illegible]	[illegible]	[illegible]	[illegible]	[illegible]	[illegible]	[illegible]	[illegible]	[illegible]	[illegible]	[illegible]	[illegible]	[illegible]	[illegible]
[illegible]	[illegible]	[illegible]	[illegible]	[illegible]	[illegible]	[illegible]	[illegible]	[illegible]	[illegible]	[illegible]	[illegible]	[illegible]	[illegible]	[illegible]	[illegible]	[illegible]	[illegible]	[illegible]	[illegible]	[illegible]	[illegible]	[illegible]	[illegible]	[illegible]	[illegible]	[illegible]
[illegible]	[illegible]	[illegible]	[illegible]	[illegible]	[illegible]	[illegible]	[illegible]	[illegible]	[illegible]	[illegible]	[illegible]	[illegible]	[illegible]	[illegible]	[illegible]	[illegible]	[illegible]	[illegible]	[illegible]	[illegible]	[illegible]	[illegible]	[illegible]	[illegible]	[illegible]	[illegible]
[illegible]	[illegible]	[illegible]	[illegible]	[illegible]	[illegible]	[illegible]	[illegible]	[illegible]	[illegible]	[illegible]	[illegible]	[illegible]	[illegible]	[illegible]	[illegible]	[illegible]	[illegible]	[illegible]	[illegible]	[illegible]	[illegible]	[illegible]	[illegible]	[illegible]	[illegible]	[illegible]
300 X 300	4T16	30	100	900	240mm X 1.6mm THK X 150mm LONG	825mm - 315mm LONG	12	310	305	40	100	50	30	1.6 X 50	15	100	4820 x 800mm	240 x 1.6mm THK X 150mm LONG	295 - 55mm	15	820 - 800mm	R6 - 55mm	15	825mm - 800mm LONG	240mm	R6 - 65mm
[illegible]	[illegible]	[illegible]	[illegible]	[illegible]	[illegible]	[illegible]	[illegible]	[illegible]	[illegible]	[illegible]	[illegible]	[illegible]	[illegible]	[illegible]	[illegible]	[illegible]	[illegible]	[illegible]	[illegible]	[illegible]	[illegible]	[illegible]	[illegible]	[illegible]	[illegible]	[illegible]
[illegible]	[illegible]	[illegible]	[illegible]	[illegible]	[illegible]	[illegible]	[illegible]	[illegible]	[illegible]	[illegible]	[illegible]	[illegible]	[illegible]	[illegible]	[illegible]	[illegible]	[illegible]	[illegible]	[illegible]	[illegible]	[illegible]	[illegible]	[illegible]	[illegible]	[illegible]	[illegible]

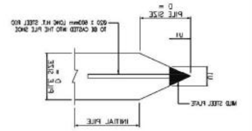

PILE SHOE TYPE J1 DETAILS

(POINTED PILE SHOE)

SCALE 1 : 10

TABLE FOR PILE SHOE TYPE J1

D NOMINAL PILE SIZE (mm)	U1
[illegible]	[illegible]
[illegible]	[illegible]
[illegible]	[illegible]
[illegible]	[illegible]
300 X 300	150
[illegible]	[illegible]
[illegible]	[illegible]

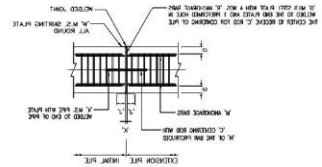

PILE JOINT DETAIL

SCALE 1 : 10

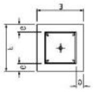

SECTION B-B

SCALE 1 : 10

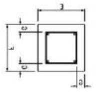

SECTION A-A

SCALE 1 : 10

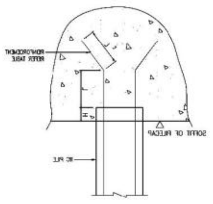

DETAIL OF PILE HEAD REINF.

SCALE 1 : 10

D NOMINAL PILE SIZE (mm)	H1 REINFORCEMENT DETAIL BAR SIZE (mm)	NOS (pcs)	L (mm)	H (mm)
[illegible]	[illegible]	[illegible]	[illegible]	[illegible]
[illegible]	[illegible]	[illegible]	[illegible]	[illegible]
[illegible]	[illegible]	[illegible]	[illegible]	[illegible]
[illegible]	[illegible]	[illegible]	[illegible]	[illegible]
300 X 300	16	4	300	100
[illegible]	[illegible]	[illegible]	[illegible]	[illegible]
[illegible]	[illegible]	[illegible]	[illegible]	[illegible]

TABLE 1 : PILES SETTING OUT SCHEDULE

STRUCTURE	SPECIFICATION OF PILE TYPE	DIAMETER (mm)	NUMBER OF PILES VERTICAL (Nos)	RAKED (Nos)	TOTAL (Nos)	GEOTECHNICAL WORKING LOAD PER PILE (kN)	MAX. ESTIMATION LENGTH OF PILE (m)
BASE TOWER FOUNDATION	SQ. R.C. PILE	300x300	9	0	9	400	24

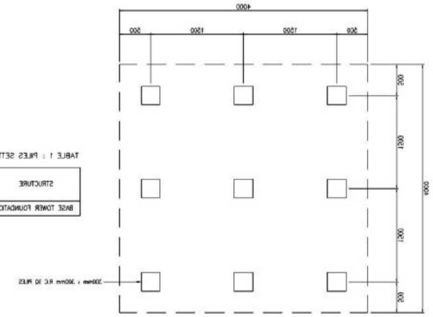

PILING SETTING OUT LAYOUT

CONSTRUCTION DRAWING

CLIENT :

KERAJAAN MALAYSIA

KEMENTERIAN SAINS, TEKNOLOGI DAN INOVASI (MOSTI)

ARAS 1-7, BLOK C4 & C5, KOMPLEKS C, PUSAT PENTADBIRAN KERAJAAN PERSEKUTUAN, WILAYAH PERSEKUTUAN 62662 PUTRAJAYA, MALAYSIA

PROJECT :

DEVELOPMENT & CONSTRUCTION OF INTERNET TRANSMISSION TOWER USING ULTRA HIGH PERFORMANCE CONCRETE (UHPC)

TITLE :

PILING SETTING OUT LAYOUT & 300mm x 300mm SQ. R.C. PILES DETAIL

ENGINEER'S SIGNATURE :

CONSULTANT :

JURUTERA PERUNDING CONCRETE TECHNOLOGY

No. 41, LAKEVIEW TWIN VILLA, LINGKARAN MERU VALLEY 2A, KLAPANG, 30020, IPOH, PERAK, MALAYSIA. TEL / FAX : +6(05) 5282813

DESIGNED BY	FAIZ	SCALE
DRAWN BY	DIANA	1 : 35
CHECKED BY	ADJ PROF IR DR VOO YEN LEI	
DATE	MARCH 2015	

REVISION :

DRAWING NO. MOSTI/JPCT/03/2015/TT/03

CONSTRUCTION DRAWING

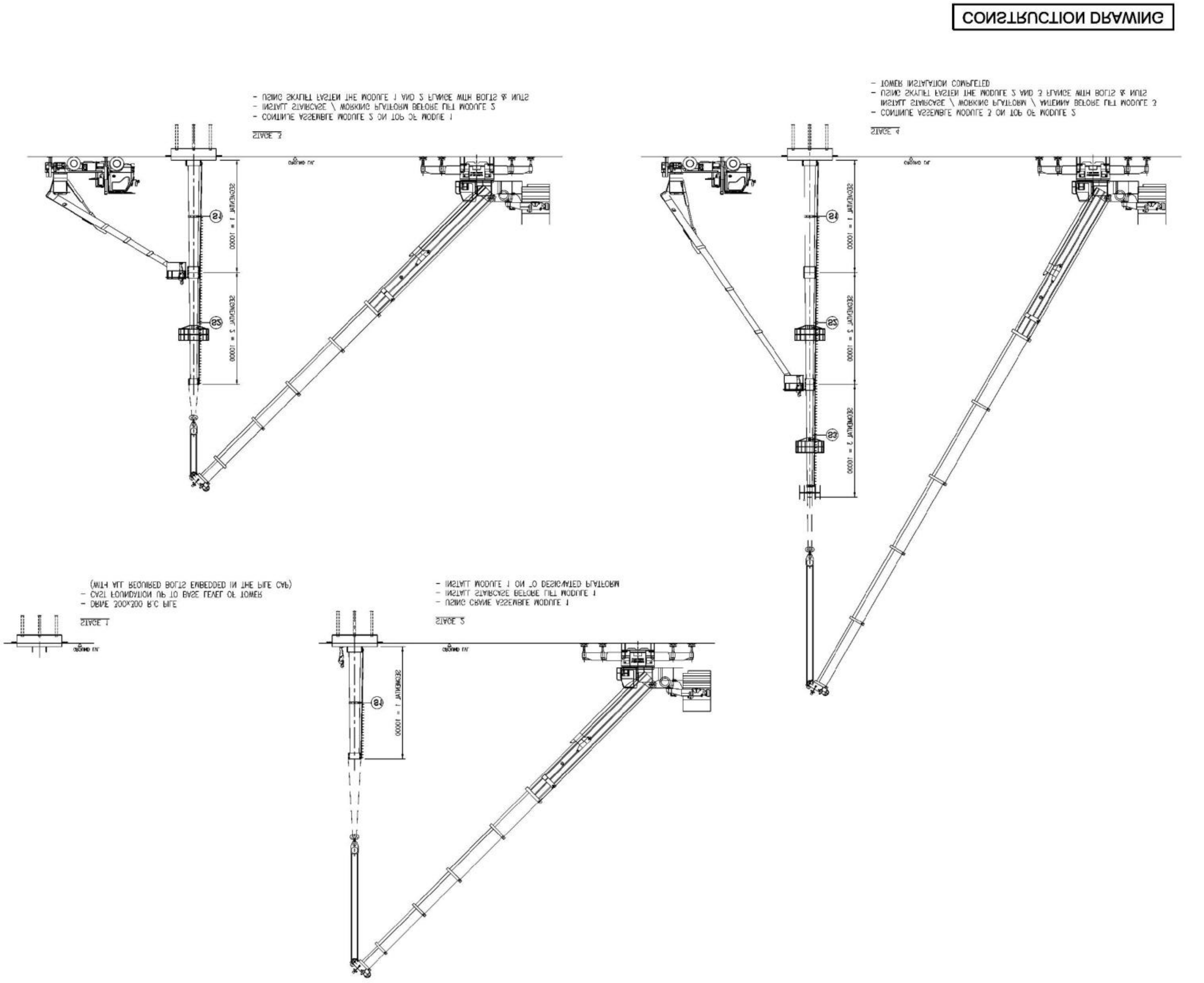

DRAWING NO.
MOSTI\JPCT\03\2015\TT\04

REVISION :

DATE	MARCH 2015	1 : 100
CHECKED BY	ADJ.PROF. IR DR VOO YEN LEI	
DRAWN BY	DIANA	
DESIGNED BY	FAIZ	SCALE

CONSULTANT :
JURUTERA PERUNDING CONCRETE TECHNOLOGY
NO. 41, LAKEVIEW TMN ALTA, LINGKARAN MERU VALLEY 2A,
JELAPANG, 30020, IPOH, PERAK, MALAYSIA.
TEL \ FAX : +6(05) 5282813

ENGINEER'S SIGNATURE :

TITLE :
TRANSMISSION TOWER
METHOD OF STATEMENT

PROJECT :
DEVELOPMENT & CONSTRUCTION OF INTERNET TRANSMISSION TOWER USING ULTRA HIGH PERFORMANCE CONCRETE (UHPC)

CLIENT :
KERAJAAN MALAYSIA
KEMENTERIAN SAINS, TEKNOLOGI DAN INOVASI (MOSTI)
