# Supplementary material for: The construction process for pre-stressed ultra high performance concrete communication tower
Source: PLoS One. 2020 Nov 4;15(11):e0238654. doi: 10.1371/journal.pone.0238654 (PMC7641448; doi:10.1371/journal.pone.0238654)
Supplement: Attachment — Submitted filename: Reviewer-Comments-Author-Reply.docx [file pone.0238654.s002.docx]

**PONE-D-19-25897**

**&**

**PONE-D-19-25897R1**

**Title: The construction process for pre-stressed ultra high performance concrete communication tower**

**Journal Requirements:**

1) Thank you for updating your data availability statement. You note that your data are available within the Supporting Information files, but no such files have been included with your submission. At this time we ask that you please upload your minimal data set as a Supporting Information file, or to a public repository such as Figshare or Dryad.

**Author reply:**

The supporting data has been prepared and uploaded accrodingly.

**Journal Requirements:**

Please ensure that you refer to Figure 11 in your text as, if accepted, production will need this reference to link the reader to the figure.

**Author reply:**

The citation to Figure 11 has been added to the text.

**Reviewer Comments** :

The submitted manuscript very interesting in presentation of new design for construction of segmental tubular section communication tower with ultra-high-performance concrete (UHPFC) material and prestress tendon to gain durability, ductility, and strength.

**Author reply:**

Thanks for review the paper and comment on manuscript.

**Reviewer Comments**:

1. The composition of the Ultra high-performance concrete (UHPFC) has to recognized in the abstract and inside the article.

**Author reply:**

The composition of UHPFRC added to abstract and also manuscript content as described in here:

The composition of Ultra high-performance concrete (UHPFC) implemented in this research is included of Cement, Densified Silica Fume (SF90), Dry Silica Fine Sand, Dry Silica Coarse Sand, Silica, Steel Fiber and Free Water.

**Reviewer Comments**:

2. Unify the unities used in the whole manuscript.

**Author reply:**

All the units throughout the manuscript have been checked and all common units are used in the body of the paper and unified it.

**Reviewer Comments**:

3. The workload of this paper is huge, and it has achieved relatively significant results, but the experimental level should be strengthened. It is important to provide with chemical composition and physical characterization of (UHPFC).

**Author reply:**

New sections are added to the manuscript as "2.1. UHPFRC Mix Design" and "2.2. Mechanics behavior of UHPFC" and all chemical composition, physical and mechanical characterization of UHPFC are demonstrated in these sections.

**Reviewer Comments**:

4. A thorough English language editing is required.

**Author reply:**

The manuscript has been reviewed and edited with English editor.

**Reviewer Comments**:

5. The authors can provide the introduction and background about other lower performance concrete and cementitious composites compared to the used (UHPFC) relative to the durability, ductility, and strength; some literatures in concern:

• H.M. Saleh, M.E. Tawfik and T.A. Bayoumi, Chemical stability of seven years aged cement–PET composite waste form containing radioactive borate waste simulates, Journal of Nuclear Materials, 411(1-3), 185-192, (2011).

• S.B. Eskander T.A. Bayoumi and H.M. Saleh, Performance of aged cement-polymer composite immobilizing borate waste simulates during flooding scenarios, Journal of Nuclear Materials 420(1-3), 175-181, (2012).

• S.B. Eskander and H.M. Saleh, Cement mortar-degraded spinney waste composite as a matrix for immobilizing some low and intermediate level radioactive wastes: Consistency under frost attack, Journal of Nuclear Materials 420(1-3), 491-496, (2012).

• H.M. Saleh and S.B. Eskander, Characterizations of mortar-degraded spinney waste composite nominated as solidifying agent for radwastes due to immersion processes, Journal of Nuclear Materials, 430(1-3), 106-113, (2012).

• H.M. Saleh, S.B. Eskander and H.M. Fahmy, Mortar composite based on wet oxidative degraded cellulosic spinney waste fibers, International Journal of Environmental Science and Technology, 11(5), 1297-1304, (2014).

• H.M. Saleh, F.A. El-Saied T.A. Salaheldin and A.A. Hezo, Macro- and Nanomaterials for Improvement of Mechanical and Physical Properties of Cement Kiln Dust-Based Composite Materials. Journal of Cleaner Production 204, 532-541, (2018).

• H.M. Saleh, S.M. El-Sheikh, E.E. Elshereafy and A.K. Essa, Mechanical and physical characterization of cement reinforced by iron slag and titanate nanofibers to produce advanced containment for radioactive waste. Construction and Building Materials 200(C), 135-145, (2019).

• H.M. Saleh, F.A. El-Saied T.A. Salaheldin and A.A. Hezo, Influence of severe climatic variability on the structural, mechanical and chemical stability of cement kiln dust-slag-nanosilica composite used forradwaste solidification. Construction and Building Materials 218(C), 556-567, (2019).

• H.M. Saleh and S.B. Eskander, Impact of water flooding on hard cement-recycled polystyrene composite immobilizing radioactive sulfate waste simulate. Construction and Building Materials 222C, 522-530, (2019).

• H.M. Saleh, S.M. El-Sheikh, E.E. Elshereafy and A.K. Essa, Performance of cement-slag-titanate nanofibers composite immobilized radioactive waste solution through frost and flooding events. Construction and Building Materials 223C, 221-232, (2019).

**Author reply:**

The latest mentioned references are reviewed and cited in the introduction part as presented in here as well.

Beside UHPFRC concrete material, many studies have been conducted about special high dense concrete materials to produce advanced containment for radioactive waste [23]. Saleh et al [24] investigated on mechanical and physical characterization of cement reinforced by iron slag and titanate nanofibers [24,27] and hard cement-recycled polystyrene composite [26] to produce advanced containment.

Also influence of severe climatic variability on mechanical and chemical stability of cement kiln dust-slag-nanosilica composite used for radwaste solidification has been investigated by Saleh et al [25, 26].

**References:**

[23] H.M. Saleh, F.A. El-Saied T.A. Salaheldin and A.A. Hezo, Macro- and Nanomaterials for Improvement of Mechanical and Physical Properties of Cement Kiln Dust-Based Composite Materials. Journal of Cleaner Production 204, 532-541, (2018).

[24] H.M. Saleh, S.M. El-Sheikh, E.E. Elshereafy and A.K. Essa, Mechanical and physical characterization of cement reinforced by iron slag and titanate nanofibers to produce advanced containment for radioactive waste. Construction and Building Materials 200(C), 135-145, (2019).

[25] H.M. Saleh, F.A. El-Saied T.A. Salaheldin and A.A. Hezo, Influence of severe climatic variability on the structural, mechanical and chemical stability of cement kiln dust-slag-nanosilica composite used forradwaste solidification. Construction and Building Materials 218(C), 556-567, (2019).

[26] H.M. Saleh and S.B. Eskander, Impact of water flooding on hard cement-recycled polystyrene composite immobilizing radioactive sulfate waste simulate. Construction and Building Materials 222C, 522-530, (2019).

[27] H.M. Saleh, S.M. El-Sheikh, E.E. Elshereafy and A.K. Essa, Performance of cement-slag-titanate nanofibers composite immobilized radioactive waste solution through frost and flooding events. Construction and Building Materials 223C, 221-232, (2019).
