# Supplementary material for: The construction process for pre-stressed ultra high performance concrete communication tower
Source: PLoS One. 2020 Nov 4;15(11):e0238654. doi: 10.1371/journal.pone.0238654 (PMC7641448; doi:10.1371/journal.pone.0238654)
Supplement: Attachment — Submitted filename: Reviewer-Comments-Author-Reply.docx [file pone.0238654.s003.docx]

**PONE-D-20-10814R1**

**Title: The construction process for pre-stressed ultra high performance concrete communication tower**

**Required Correction 1)**

Please ensure that you refer to Table 4,5 and 7 in your text as, if accepted, production will need this reference to link the reader to the Table.

**Author reply:**

All tables are cited in the manuscript.

**Required Correction 2)**

We note that you currently have two Tables in your manuscript titled as Table 1 and two Tables titled as Table 2.

The first "Table 1: Mix design for UHPFRC used for construction of communication tower  - and the second "Table 1: Mix Design for UHPFRC With Micro and Hooked Ends Steel Fibers.
The first "Table 2: The material properties of steel reinforcement used - and the second "Table 2: Mechanical properties of UHPFC material. So that these tables can be differentiated can you please update the Table title numbering and the in-text citations to them accordingly.

**Author reply:**

Thanks for the comment and apologize for the typo mistakes. All table numbers are sorted carefully.

**Required Correction 3)**

Based on the information you have provided us, we propose the following funding and competing interests statements for your approval:

Funding:
"This research receives support from Dura Technology Sdn. Bhd. ([https://dura.com.my](https://dura.com.my/)) via Dr. Voo Yen Lai with grant project with Vot No. 6300195. Dura Technology Sdn. Bhd. received this fund from Ministry of Science Technology & Innovation, Malaysia ([https://www.mestecc.gov.my](https://www.mestecc.gov.my/)) for the project entitled as “Development and construction of Internet Transmission Tower Using Ultra High Performance Concrete”. Their help and support are gratefully acknowledged. The sponsors or funders have no any role in the study design, data collection and analysis, decision to publish, or preparation of the manuscript."

Competing interests:

This research receives support from Dura Technology Sdn. Bhd. ([https://dura.com.my](https://dura.com.my/)). This does not alter our adherence to PLOS ONE policies on sharing data and materials.

Can you please confirm the above statement is both complete and correct? With your approval we will update these statements on your behalf. If you approve of them, please make this clear in your cover letter or author comments.

**Author reply:**

Yes, I am confirmed the above statements. This statements are added in the cover letter.

**Required Correction 4)** We note your current Data Availability statement: "The data underlying the results presented in the study are available from Corresponding Author."

Please note that PLOS journals require authors to make all minimal data underlying the findings described in their manuscript fully available without restriction at the time of publication, with rare ethical or legal exceptions: <https://journals.plos.org/plosone/s/data-availability>.

PLOS ONE describes the minimal data set as that which is used to reach the conclusions drawn in the manuscript with related metadata and methods, and any additional data required to replicate the reported study findings in their entirety. This may include:
- The values behind the means, standard deviations and other measures reported
- The values used to build graphs
- The points extracted from images for analysis

Please provide the name of the ethics committee or Institutional Review Board that is imposing sharing restrictions on your de-identified minimal data set and the grounds for restriction (e.g. contains sensitive identifying information).

**Author reply:**

There is no restriction on presented data in this paper and all data already provided within manuscript.
